# Do Wet-Dry Ratio and Fe-Mn System Affect Oxidation-Reduction Potential Nonlinearly in the Subsurface Wastewater Infiltration Systems?

**DOI:** 10.3390/ijerph15122790

**Published:** 2018-12-09

**Authors:** Xiaorong Zhang, Haibo Li, Yinghua Li, Fei Guo, Zhongxin Yang, Jianing Bai

**Affiliations:** School of Resources and Civil Engineering, Northeastern University, Shenyang 110819, China; iamlhb@126.com (X.Z.); m18537919311@163.com (F.G.); zxyoung825@163.com (Z.Y.); 18841249919@163.com (J.B.)

**Keywords:** wet-dry ratios, ORP, SWIS, redox microenvironment, Fe-Mn system

## Abstract

To understand characteristics of on-line oxidation-reduction potential (ORP) in a subsurface wastewater infiltration system (SWIS) under different intermittent influent conditions, ORP among five matrix depths at wet-dry ratios (R_wd_s) of 2:1, 1:1 and 1:2 with a hydraulic load of 0.10 m^3^·(m^2^·d)^−1^ were monitored. Results showed that the optimal R_wd_ for the SWIS was 1:1. In that case, ORP at 40 and 65 cm depths changed significantly, by 529 mV and 261 mV, respectively, from the inflow period to the dry period, which was conducive to the recovery of the oxidation environment. It was concluded that ORP varied nonlinearly in strongly aerobic and hypoxic environment. Wastewater was fed into the SWIS at 80 cm and dissolved oxygen diffused at the initial period of one cycle. As a consequence, ORP at 65 cm increased with water content increasing. However, ORP at 40 and 95 cm displayed inverse trends. Moreover, results showed that ORP decreased with Fe^2+^ and Mn^2+^ increasing under aerobic conditions (*p* < 0.05) because Fe^2+^ and Mn^2+^ moved with wastewater flow. Effluent met reuse requirements and no clogging was found in the SWIS during the operation.

## 1. Introduction

Facing with multiple challenges of energy shortage, resource depletion and environmental pollution, sustainability is considered to be a pivotal criterion and driving force to further advancement [1]. There are many green technologies to achieve sustainable wastewater treatment, such as microbial fuel cells-centered technologies and membrane technologies, which can be applied in textile and saline water [2]. Compared with membrane technologies, which generate membrane wastewater during the membrane fabrication processes, subsurface wastewater infiltration systems (SWISs), as representatives of decentralized sewage treatment, are considered to be satisfactory biological reactors with low construction and operation expenses, satisfactory pollutant removal performances and lower cost of maintenance [3,4]. Furthermore, SWISs meet water reclamation and reuse requirements. In contrast to soil self-purification, sewage is fed into to a certain depth of SWISs and infiltrated to oxic areas evenly by capillary and gravity forces [5]. In the case of this condition, pollutants are removed by a series of physical, chemical and biological reactions. Fayyad et al. [6] claimed that nitrification and denitrification were sensitive to redox potential (Eh) at different matrix depths in soil. However, SWISs are unstable systems because metallic ions, such as ferrous and manganese ions, move at any time. As a consequence, the whole system is under an active condition when wastewater is flowing. The microenvironment can also thus be more active. As a consequence, it is inappropriate to use soil Eh to estimate conditions of SWISs. Conversely, using on-line oxidation-reduction potential (ORP) to monitor the microenvironment and ensure the conditions of pollutant treatments has been proven to be useful technology for controlling SWISs [7]. ORP obtained helps identify and control removal of biological nutrient and hexavalent chromium [8]. Kuroda et al. [9] pointed out that nitrification, denitrification and phosphorous removal needed adequate ORP conditions in order to achieve TN (total nitrogen) and TP (total phosphorous) discharge standards. Husson [10] demonstrated that ORP could provide essential information to characterize soil conditions. Furthermore, ORP, an indicator taking the place of dissolved oxygen (DO), can characterize the state of the redox microenvironment by establishing equilibrium potential [11]. Several types of operations for removing pollutants by means of SWISs have been carried out. In comparison to continuous operations, different wet-dry alternation operations in SWISs are alternative means of enhancing reoxygenation and the nitrification processes [12]. Suitable R_wd_ (the time ratio of inflow period to dry period) is considered to be an essential factor for improving the function of SWISs. Sun et al. [13] pointed out that wet-dry alternation operations could enhance the removal rates of organic pollutants and ammonia nitrogen. Well-drained soil and gravel rock type allow faster movement of groundwater. In that case, oxic groundwater conditions with low denitrification potential and a high nitrogen loading rate could be obtained [14]. In addition, the study found that TN removal and low N_2_O emission could be achieved under intermittent aeration with an influent surface organic loading of 21.2 and 33.8 g COD (Chemical Oxygen Demand) (m^2^·d)^−1^ in the SWIS [15]. However, thus far, many researchers have focused on the removal of pollutants but ignored the mechanism of the microenvironment. In addition, existing observations do not have continuity and reliability, so previous studies were unable to observe the momentary changes of ORP in the SWIS [16]. Weil and Brady [17] pointed out that ORP is only related to matrix depths and dissolved oxygen, which was not subjected to the influence from intermittent operations and hydraulic loads. The theory is that ORP at a certain matrix depth has the overall consistency with no local fluctuations. Nevertheless, the specific changes in different matrix depths are extremely complicated because the microenvironment changes in the SWIS. This theory has not been confirmed by experiments and lacks quantitative expression of how intermittent operations influenced ORP at different matrix depths in SWISs [18]. Moreover, the effects on ORP of iron (Fe) and manganese (Mn) ions migrating among different depths were also unclear.

Aiming to observe and learn the regulations of ORP at different matrix depths, determine satisfactory conditions for enhancing the denitrification and nitrification in the SWISs and make full use of values of the wastewater, R_wd_s were changed and the hydraulic load was kept constant. Temperature was kept between 22 and 26 °C. The combination of on-line ORP and different R_wd_s in the SWISs is proposed for understanding the variations of the redox microenvironment in more detail. The optimal R_wd_ at different matrix depths was selected so that the SWIS ran well. Additionally, migrations of Fe^2+^ and Mn^2+^ in the SWIS under different R_wd_s were considered and their impacts on the ORP are discussed.

## 2. Materials and Methods

### 2.1. SWIS Description

**Figure 1:**
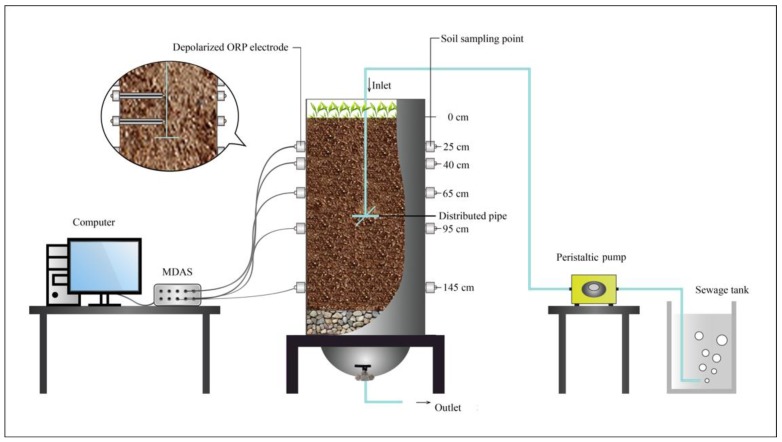
Schematic diagram of the subsurface wastewater infiltration system (SWIS). ORP: oxidation-reduction potential; MDAS: multi-channel data acquisition system.

Figure 1 briefly describes the SWIS. The major part of the SWIS was a soil column in plexiglass (60 cm in width, 100 cm in length, with a depth of 200 cm). In order to fill the matrix conveniently, the plexiglass was divided into an upper (60 cm), middle (70 cm) and lower part (70 cm) by flanges. Depolarization ORP detection probes were placed in advance in the center of the SWIS at 25 cm, 40 cm, 65 cm, 95 cm and 145 cm matrix depths and there were sampling points at each matrix depth for collections of soil samples. Wastewater was fed into the SWIS via a distributing pipe (100 cm length and 20 mm diameter), which was wrapped with sand and placed at the 80 cm matrix depth over a Niimi slot (a kind of anaerobic slot). Treated water was gathered at the bottom of the system. Statistics were collected by multi-channel data acquisition system (MDAS), which recorded instantaneous changes of on-line ORP at different R_wd_s. Data was transmitted to the sensors in the form of electrical signals and collected by computer. Wastewater was fed into the system at a constant hydraulic load rate of 0.10 m^3^·(m^2^·d)^−1^ by a peristaltic pump (Prefluid BT100-YZ15) which was controlled by a timer switch. A sewage tank was utilized to contain wastewater. ORP probes were manufactured by Nanjing Soil Research Institute in China.

### 2.2. Matrix

Three kinds of matrix were chosen and mixed in volume for the SWIS (70% brown soil, 20% coal slag and 10% sand). Coal slag and sand were selected to improve the permeability of the SWIS. At the bottom of the column, gravel was selected and tiled about 20 cm thick to avoid clogging. Clean water should infiltrate evenly when filling the matrix, so that the SWIS compacted naturally. If there was a large volume of space in the SWIS, sensitivity of ORP probes would decrease and the on-line ORP would not be accurate. Once the matrix was filled to the depths of the sampling points, ORP probes were inserted in advance after corrections. Brown soil was collected 0–20 cm below the soil surface from the west of Shenyang in Liaoning Province. Coal slag was obtained from a boiler room in Shenyang, with dimensions of 1 mm to 5 mm in diameter. In addition, sand and gravel with diameter of 1 mm to 5 mm were purchased for the SWIS.

### 2.3. Matrix, Water Quality and Data Analysis

Physical and chemical properties of the matrix were analyzed. The organic matter was measured after removing inorganic matter by 25% hydrochloric acid [19]. Permeability and particle size distribution were investigated by the single-ring infiltrometer method [20] and a particle size meter, respectively. In addition, porosity was computed referring to Grossman and Reinsch’s method using bulk density data [21]. Hydraulic conductivity was measured using a method from Reynolds et al. [22]. Specific properties of the mixed matrix were shown in Table 1.

Additionally, raw wastewater which was obtained from the campus in Shenyang after pretreatment was investigated. This could fluctuate with the living habits of the people in different seasons. Furthermore, treated wastewater was collected. COD, BOD_5_ (Biochemical oxygen demand), NH_4_^+^-N (Ammonia nitrogen), TN and TP of the wastewater were analyzed every week based on the standard methods [23]. Results are shown in Table 2.

Origin 2017 and MATLAB 2014a were employed to analyze the relationships among ORP, R_wd_s and the contents of Fe^2+^ and Mn^2+^. ORP was collected by MDAS. SPSS 19.0 was used to evaluate whether there was a significant difference in ORP at different R_wd_s according to the experimental results. The schematic diagram of the SWIS was drawn using Photoshop 2016.

### 2.4. Experiments

#### 2.4.1. ORP in Different R_wd_s

To study the effects of R_wd_s on matrix microenvironment specifically, a SWIS was set up and ORP at five matrix depths (25, 40, 65, 95, and 145 cm) was collected by MDAS every 30 min. The study took place over a total of 154 days and included 2 runs. During the first run, wastewater was fed into the SWIS with a hydraulic load of 0.10 m^3^·(m^2^·d)^−1^ at the R_wd_ of 2:1 (8 h:4 h), 1:1 (8 h:8 h) and 1:2 (4 h:8 h), including an inflow period of 8 h, 4 h, 4 h with a dry period of 4 h, 8 h and 8 h in one cycle, respectively. During the experiments, the hydraulic load was kept constant by a peristaltic pump. Before the experiments were carried out, the experimental device was debugged for 2 months so that the SWIS was stable, and on-line ORP was monitored for 1 month under each R_wd_. In order to make each operation condition independent, wastewater was not allowed for 48 h in the SWIS. Therefore, each experiment ran for 32 days. ORP at 5 matrix depths was investigated. Fluctuations of ORP were analyzed in detail. The optimal R_wd_ was suggested. During the second run, the contents of Fe^2+^ and Mn^2+^ from five matrix depths were determined during the inflow periods and then their effects on ORP were analyzed. The optimal R_wd_ was also selected. Data was sorted and analyzed after the experiments ended. Triplicate readings for each sampling point were recorded and averages were used for analyzing. Because temperature was controlled between 22 °C and 26 °C in the laboratory, the effects of temperature on ORP and Fe^2+^ and Mn^2+^ content were neglected.

#### 2.4.2. Measurements of the Contents of Fe^2+^ in the Matrix

2–5 g samples from different matrix depths in the SWIS were collected and put into aluminum sulfate solution (0.1 mol·L^−1^, pH = 2.5) immediately each time with a volume ratio of 1:20. New mixed samples were shaken for 30 min at 100 rpm by shaker and then centrifuged for 3 min at 4000 rpm in the centrifuge.

A quantity of 5 mL supernatant was fed into a 50-mL colorimetric cube. In order to adjust pH to 5, 8 mL sodium acetate solution (100 g·L^−1^) was added into the clean cube. A quantity of 10 mL phenanthroline solution was put into the mixture solution, which was then distilled to 50 mL. The contents of Fe^2+^ was measured at 560 nm by a UV spectrophotometer (Model: DR 6000, HACH, Loveland, CO, USA)

#### 2.4.3. Measurements of the Contents of Mn^2+^ in the Matrix

A quantity of 10 mL supernatant was fed into a clean colorimetric cube (50 mL) and then 2.5 mL H_2_SO_4_ (1:1 in volume), 1.5 mL H_3_PO_4_, 0.25 g KIO_4_ and 16 mL deionized water were added. After heating in a water bath for 30 min, the solution was distilled to 50 mL. The contents of Mn^2+^ were measured at 530 nm by a UV spectrophotometer (Model: DR 6000, HACH, Loveland, CO, USA).

Data was sorted and analyzed after the experiments.

## 3. Results and Discussion

### 3.1. ORP under Three R_wd_s in the SWIS

**Figure 2:**
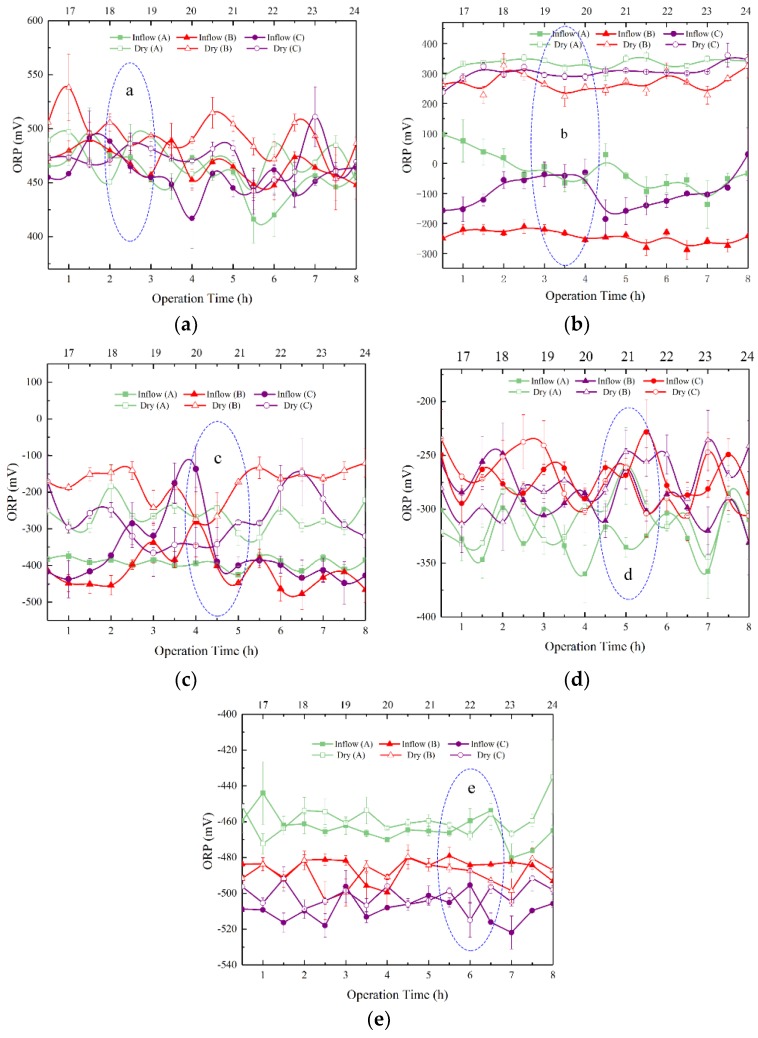
ORP variations with operation time at five matrix depths and three R_wd_s. The bottom *X*-axis represents the operation time at the inflow periods and the upper *X*-axis represents the operation time at the dry periods. The *Y*-axis represents the ORP values. ORP values and their standard deviation at the inflow period and dry period at the R_wd_ of 2:1, 1:1 and 1:2 are shown as Inflow (A) and Dry (A), Inflow (B) and Dry (B), Inflow (C) and Dry (C), respectively. ORP variations with the operation time are at the R_wd_ of 2:1, 1:1 and 1:2 in a matrix depth of: (**a**) 25 cm; (**b**) 40 cm; (**c**) 65 cm; (**d**) 95 cm; and (**e**) 145 cm.

Figure 2 shows ORP at the 25, 40, 65, 95, and 145 cm depths at the R_wd_ of 2:1, 1:1 and 1:2. The bottom *X*-axis represents the operation time at the inflow periods and the upper *X*-axis represents the operation time at the dry periods. The *Y*-axis represents the ORP. Results proved that ORP decreased with depths increasing. A similar trend was found by Zhang [24]. Under the wet-dry alternation operations, ORP at a certain depth in the SWIS fluctuated periodically at the R_wd_ of 2:1, 1:1 and 1:2.

The study found that ORP in the initial case was significant different (*p* < 0.05) under three R_wd_s. The standard deviation (SD) is shown in the form of error bars. SD obtained by calculating the positive and negative square roots of the variances can effectively reflect the degree of dispersion of a data set. According to the SD, few big fluctuations were found, which suggested that the system was stable to some extent. According to the theory of the redox environment of Whisler [25], the whole SWIS was considered to be divided into four areas: aerobic area, hypoxic area, common anaerobic area and complete anaerobic area. In addition, Lance put forward the view that ORP in the aerobic, hypoxic and completely anaerobic environments were above 300 mV, 300 mV to −200 mV, and below −200 mV, respectively [26].

As shown in Figure 2a, ORP between the inflow and dry periods at the 25 cm matrix depth was basically unaffected under three R_wd_s (*p* > 0.05). Lance put forward the view that ORP under the aerobic environment was above 300 mV [26]. Given that ORP fluctuated between 416 mV and 525 mV, 437 mV and 538 mV, and 408 mV and 525 mV at the R_wd_ of 2:1, 1:1 and 1:2, respectively, it can be concluded that R_wd_s had little effect on the completely aerobic zone. ORP during the inflow periods was a little higher than that during the dry periods. The main reason was that pore water occupied the position that belonged to pore air in the middle depths. Therefore, oxygen diffused upward and ORP increased during the inflow periods. At the R_wd_ of 2:1, 1:1 and 1:2, results showed that ORP at the 25 cm matrix depth fluctuated irregularly. The biggest fluctuation of ORP from the inflow period to the dry period was 117 mV, which was found at the R_wd_ of 1:1. The major reason was that the area observed was close to the surface of the SWIS, so that oxygen moved flexibly. Because the porosity of the mixed matrix was high, capillary force was slight. What’s more, capillary force decreased compared to that at the initial condition as the water content increased. Therefore, ORP at the 25 cm matrix depth was in the aerobic state all the time.

The ORP decreased during the inflow periods and rose during the dry periods at the 40 cm matrix depth under three R_wd_s. Compared with Figure 2a, since wet–dry alternation operations were changed, ORP at the 40 cm matrix depth (Figure 2b) decreased sharply from 359 mV to −138 mV, 326 mV to −288 mV, and 361 mV to −185 mV at the R_wd_ of 2:1, 1:1 and 1:2, respectively. The biggest fluctuation of ORP from the inflow period to the dry period, of 614 mV, was found at the R_wd_ of 1:1. During the dry periods, ORP under three R_wd_s was about 300 mV. As a consequence, the system was almost under the aerobic environment. Nevertheless, results showed that the environment became hypoxic and even anaerobic. Moreover, because the time for effluent at the R_wd_ of 2:1 was shorter than at the R_wd_ of 1:1 and 1:2, it took longer for the microenvironment to stabilize under the hypoxic state. Fayyad et al. pointed out that the nitrification was prone to occur when ORP was between 200 and 400 mV [6]. Denitrification would occur when the ORP was between −100 mV and 100 mV [27]. Furthermore, the denitrification reaction could be restricted if the ORP increased above 400 mV [28]. Ong et al. [29] and Yang et al. [30] declared that abundant oxygen elevated the performance of aerobic biochemical oxidation. Moreover, the microenvironment was in stable aerobic and anaerobic states during the inflow and dry periods, respectively. Therefore, it can be deduced that the activity of the redox microenvironment at the 40 cm matrix depth of the SWIS was strong and pollutants were removed efficiently in this area.

Figure 2c shows that ORP and its fluctuations decreased compared to those at the 40 cm matrix depth. Results showed that the hypoxic and anaerobic environments appeared alternately between the dry periods and the inflow periods in the SWIS. Similarly, the biggest fluctuation of ORP, which decreased to −476 mV during the inflow period and increased to −120 mV during the dry period, was found at the R_wd_ of 1:1. Therefore, denitrification was more apparent than nitrification in this area according to the ORP, indicating it was beneficial for the microenvironment. The SD was still obvious and large at the 65 cm matrix depth, suggesting that the SWIS designed in this study was an active system. At the end of the inflow period, the flow moved by horizontal diffusion.

With the matrix depths increasing, the SWIS stabilized gradually and became an anaerobic environment, maintaining −300 mV up and down with slight fluctuations at the 95 cm matrix depth (Figure 2d). SDs began to increase compared to those at the 40 and 65 cm matrix depths, which indicated that the redox environment was unsteady and denitrification reactions occurred frequently. ORP at the R_wd_ of 1:1 increased slightly and eventually remained at −200 mV. The main reason for this phenomenon was that the water saturated the middle layer, and even flooded it, with the increasing inflow. ORP at the R_wd_ of 2:1 was relatively small. The speed of seepage of dissolved oxygen accelerated significantly when the retention time of the wastewater during one cycle in the SWIS was further shortened by four hours. Thus, it could be seen that the anaerobic environment was sensitive to the operation conditions at the R_wd_ of 1:2.

At the bottom of the SWIS, it was obvious that ORP at the 145 cm matrix depth decreased with the R_wd_s decreasing under the completely anaerobic environment. Figure 2e shows that the ORP at the 145 cm matrix depth was relatively stable, followed by −460 mV, −490 mV and −510 mV up and down at the R_wd_ of 2:1, 1:1 and 1:2, respectively. The main reason was that the matrix layer at the bottom of the SWIS was not exposed to oxygen sufficiently [31].

**Figure 3:**
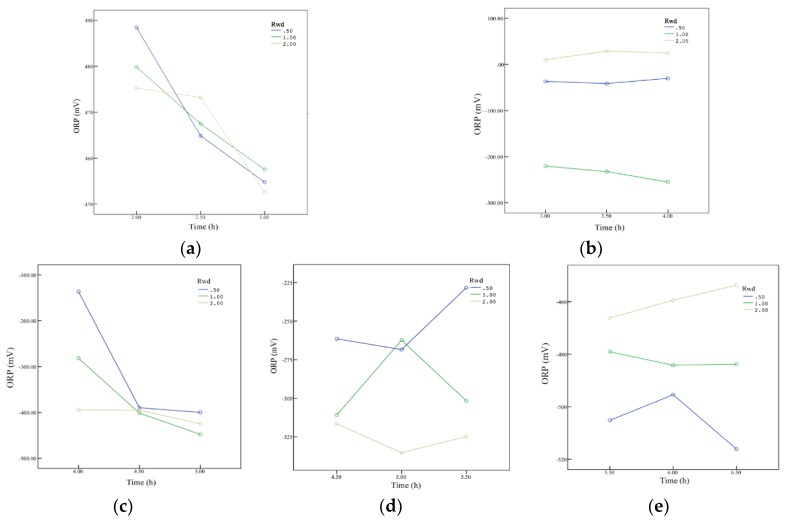
Interaction analysis between the operation time and R_wd_s in five matrix depths in the SWIS, where data analyzed in Figure 3a–e is drawn from the blue dotted areas of Figure 2a–e, respectively. Interaction analysis between the operation time and R_wd_s at depths of: (**a**) 25 cm; (**b**) 40 cm; (**c**) 65 cm; (**d**) 95 cm; and (**e**) 145 cm.

As can be seen from Figure 3, the ORP in each matrix depth changed with the synergistic effect between operation time and the R_wd_s. Figure 3a–e represents the dotted area responding to Figure 2a–e, respectively. Among Figure 3a–e, ORP at the 40 cm matrix depth (Figure 3b) underwent little synergy with minor variations. ORP at the other matrix depths, especially 65 cm (Figure 3c) and 95 cm (Figure 3d), were sensitive to operation time and R_wd_s. Consistently, these data were collected at the bottom of the distributing pipe. Because of the good permeability of the matrix in the SWIS, water content in these two areas was high and the flow of moisture in these areas was obvious. As a consequence, water contents fluctuated in these areas in a short time. Due to changing R_wd_s, the flow rates during the inflow periods were different, which greatly controlled the ranges of water content. Therefore, ORP changed under both the effects of operation time and R_wd_s. Systematic approaches for allowing simultaneous variations of more than one factor (e. g. hydraulic load, temperature and R_wd_) and evaluating the cause and effect relationships could contribute to understanding and presenting an optimal application pattern of the SWIS [32]. Sensitivity analysis [33] could also be used to determine the critical factor for satisfactory SWISs.

In summary, the sequence of descending speed of ORP changes was 2:1, 1:1, 1:2, which was consistent with the increase of inflow per unit of time.

It was clearly showed that R_wd_s had little impact on ORP under the hypoxic and the totally aerobic situations (*p* < 0.05). Conversely, the redox environment became more complicated at the matrix depth from 40 to 95 cm with R_wd_s changing. Figure 2 shows that ORP fluctuated at the same time and the R_wd_s broke the linear distribution. ORP was sensitive to environmental changes. The strong fluctuations of ORP might indicate that microorganisms were more active [34].

Overall, the results showed that ORP under three R_wd_s fluctuated greatly in the SWIS, especially at the 40 to 95 cm matrix depth. SWIS contained aerobic, hypoxic, anaerobic and completely anaerobic areas. The obvious periodic variations proved that the SWIS was strongly stable and had great ability to adjust the microenvironment at the 40 cm matrix depth. The performances of the SWIS were satisfactory according to the experimental results of outflow quality.

**Figure 4:**
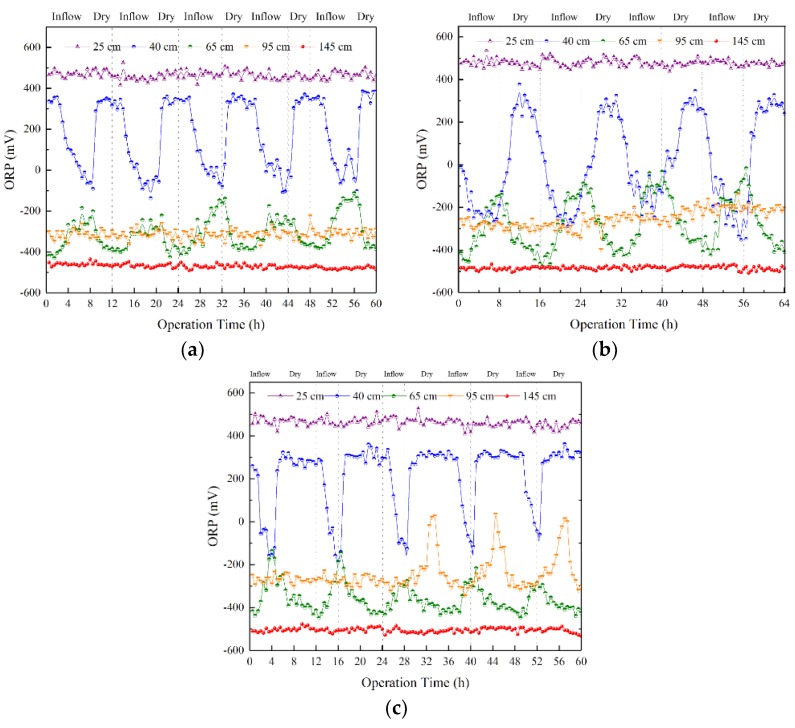
ORP variations in five matrix depths with R_wd_s. The bottom *X*-axis indicates the operation time and the upper *X*-axis indicates the wetting and drying status of the simulation system. The *Y*-axis indicates the ORP values. Data was recorded every 30 min. ORP is at in five matrix depths in the SWIS at the R_wd_ of: (**a**) 2:1; (**b**) 1:1; and (**c**) 1:2.

Figure 4 shows the fluctuations of ORP in the SWIS at the R_wd_ of 2:1, 1:1 and 1:2. In terms of Figure 2, ORP at the 40 and 65 cm matrix depths clearly overlapped. Different from basic principles of soil science [17], it indicated that the original environment changed. The frequency fluctuations of ORP at five matrix depths were also different with R_wd_s changing.

According to Figure 4a, the internal environment of the SWIS was sensitive at the R_wd_ of 2:1, which could become a hypoxic environment from an aerobic environment at the 40 cm matrix depth. Results showed that ORP could decline to below −100 mV at the end of the inflow period and then rapidly increase to above 350 mV. Furthermore, hypoxic and anaerobic environments occurred alternately at the 65 cm matrix depths according to ORP. However, results showed that ORP increased during the inflow period and decreased during the dry period at 95 cm, which was contrary to behavior at 65 cm. The reason was that the content of CO_2_ might increase because of microorganism respiration. In addition, it took some time for wastewater to reach the 65 cm matrix depth. It was obvious that the nitrification reaction occurred at above 65 cm and the denitrification reaction only occurred below 65 cm.

The more sharply the ORP fluctuated, the more intense the redox reactions [35]. ORP at the 40 and 65 cm matrix depths changed significantly, especially at the R_wd_ of 1:1 (Figure 4b). Furthermore, ORP at different depths began to overlap. ORP was almost −200 mV from 40 cm to 95 cm matrix depths when the SWIS ran for 4 h, 20 h, 36 h, and 52 h. This indicated that denitrification occurred when the wastewater was fed into the SWIS for 4 h below the 40 cm matrix depth. As a consequence, the removal ability of the SWIS could be improved. The maximum ORP at the 65 cm matrix depth at the R_wd_ of 1:1 increased by 100 mV compared to that at the R_wd_ of 2:1.

Compared with ORP changes at the R_wd_ of 1:1 in the SWIS, ORP fluctuations became smaller at the 40 cm matrix depth at the R_wd_ of 1:2 (Figure 4c). The biggest ORP at the R_wd_ of 1:2 was smaller than at the R_wd_ of 1:1. The reason was that the yield of wastewater at the R_wd_ of 1:2 was less than that of 1:1, and the time of inflow was so short that capillary action failed to fully develop above the 40 cm matrix depth. Similarly, time of effluent was longer than that of inflow so the matrix failed to recover for the next run. Actually, the redox environment had an important effect on the removal of organic matters [36]. The decomposition rate of organic matter in the aerobic layer was much higher than that in the anaerobic layers. Studies have shown that the content variations of nitrate nitrogen in different reaction areas of the SWIS are related to ORP [6]. Kong et al. pointed that the organic pollutants were affected by the oxygen content of the matrix, and oxygen convection and diffusion at the 100 cm matrix depth obtained purification [37]. Therefore, the SWIS performed steadily ay the 95 cm matrix depth.

In terms of Figure 4, a non-linear trend of ORP changes is obvious. Contrary to ORP collected from 40 and 95 cm depths, ORP increased at first and then decreased at the 65 cm depth. Denitrification mainly occurred below the 65 cm matrix depth when the wastewater was fed into the SWIS for 4 h with a R_wd_ of 1:1. Additionally, nitrification mainly occurred above the 40 cm matrix depth.

### 3.2. Effects of Fe-Mn Environment on ORP

**Figure 5:**
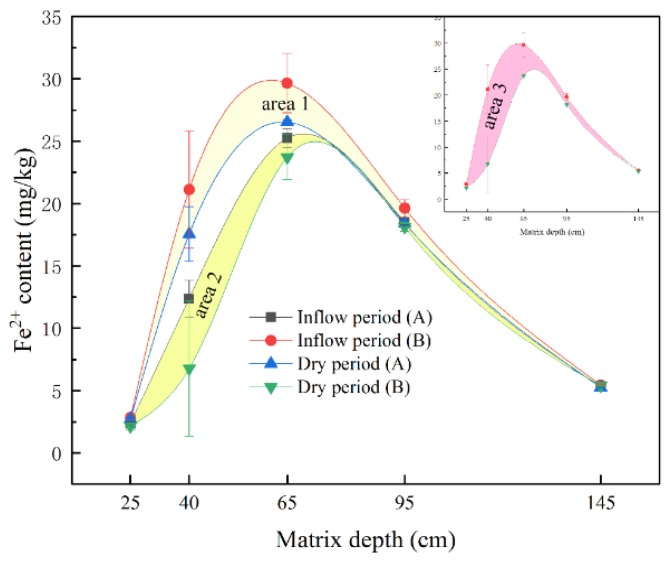
Changes of Fe^2+^ content under wet-dry alternation operations at matrix depths. Inflow period (A) and inflow period (B) describe the Fe^2+^ content when the duration of the inflow period was 4 h and 8 h, respectively. Dry period (A) and dry period (B) describe the Fe^2+^ content when the duration of the drying period was 4 h and 8 h, respectively. Area 1 to area 3 show the amplitude of Fe^2+^ variations at the R_wd_ of 2:1, 1:2 and 1:1, respectively.

As slag and other modified materials were added into the SWIS, redox reactions under the wet-dry alternation conditions were complicated. The ferromanganese system was also sensitive to the environment [18]. Figure 5 shows the contents of Fe^2+^ with time extended at different depths, where Dry period (A) and Dry period (B) show Fe^2+^ with an outflow period of 4 h and 8 h, respectively. Wet period (A) and Wet period (B) show Fe^2+^ when the length of the inflow period was 4 h and 8 h, respectively. Results showed that Fe^2+^ was sensitive to the redox microenvironment. Fe^2+^ increased at first and then decreased with depth. The trend of Fe^2+^ variations was similar to a study in deep colluvial soil by Mareckova et al. [38]. Furthermore, the contents of Fe^2+^ varied considerably at the 40 and 65 cm matrix depths. The reason was that Fe^2+^ generated radical oxygen species which could effectively enhance the decomposition rate of organic matter, making this area unsteady [39,40]. The maximum and minimum Fe^2+^ found at 40 cm matrix depth was 21.12 mg·kg^−1^ (Inflow period (B)) and 6.78 mg·kg^−1^ (Dry period (B)). The maximum and minimum Fe^2+^ found at 65 cm matrix depth was 29.65 mg·kg^−1^ (Inflow period (B)) and 23.72 mg·kg^−1^ (Dry period (B)). It can be concluded that the contents of Fe^2+^ were the highest at each matrix depth when the wastewater was fed into the SWIS for 8 h. The main reason was that oxygen concentration declined. Low oxygen contributed to Fe^3+^ converting into Fe^2+^ [41]. Oxidation–reduction reactions were important in the solubilization and precipitation of iron. According to the data shown in Figure 2 and Figure 4, it is known that nitrification and denitrification could occur frequently in the SWIS. Thus, Fe^2+^ are active valence metals that participate in equilibrium of precipitation, dissolution and redox reactions. As a result of seepage, Fe^2+^ could enhance microbial decomposition by serving as terminal electron acceptors in anoxic soils with water infiltration or diffusion. In addition, ions combined organic molecules to impede microbial decomposition [42]. Therefore, the impacts of Fe^2+^ on ORP were extremely important. Because R_wd_s changed from 2:1 to 1:2, some Fe^2+^ generated during the inflow period (A) were included in the dry period (B). As a consequence, Fe^2+^ contents during the dry period (A) were higher than those during the inflow period (B). Area 1, area 2 and area 3 indicate the trends of fluctuation changes of Fe^2+^ at the R_wd_ of 2:1, 1:2 and 1:1, respectively. Fe^2+^ fluctuated clearly from 40 to 95 cm depths at the R_wd_ of 2:1. Similarly, Fe^2+^ fluctuated intensely from 25 to 65 cm depths at the R_wd_ of 1:2 and 1:1. The widest amplitude of Fe^2+^ fluctuation was found in area 3. That is, the contents of Fe^2+^ changed significantly at the R_wd_ of 1:1. It can be concluded that Fe^2+^ moved actively, especially from 40 to 65 cm depths. ORP created good microbial and chemical conditions for iron redox. It can be concluded from the results that Fe^2+^ played a leading role from −400 mV to 350 mV. Furthermore, Fe^2+^ played a critical role in phosphorus removal, in the precipitation process with iron salts. P cycling can be strongly coupled with sediment iron under redox conditions. If Fe^3+^ was reduced, more PO_4_^3−^ was released [43]. In that case, P removal decreased, indicating that the precipitation process played a serious role in improving P removal according to Table 2. In summary, Fe^2+^ was one of the factors that influenced the nonlinear phenomenon of matrix redox potential between the 40 and 65 cm matrix depths. Under the wet–dry alternation conditions, the effects of iron redox in the matrix were prominent.

Mn^2+^ action in the matrix was significant and also further influenced ORP (Table 3). The content of Mn^2+^ at five matrix depths during the inflow periods at the R_wd_ of 1:1 are shown. Table 3 shows that a smaller amount of Mn^2+^ existed than that of Fe^2+^ in the SWIS. ORP increased with Mn^2+^ increasing (*p* < 0.05). Because the wastewater was pumped into the middle of the SWIS, weak oxidation was suitable for manganese bacteria in the middle of the matrix. Mn^2+^ in the matrix could be converted into Mn^4+^ and transitional Mn^3+^ when oxidant (mainly O_2_) was consumed, and would eventually be oxidized to Mn^4+^ to form high-priced manganese oxides. As a consequence, Mn^2+^ also decreased with ORP decreasing. On the other hand, Mn^4+^ was reduced to soluble Mn^2+^, which could exude from the matrix or dissolve in pore water under anaerobic conditions [44]. As a consequence, ORP was reduced. However, Mn^2+^ increased at first and then decreased with increasing matrix depth in the SWIS. It can be concluded that abundant oxygen was brought into the SWIS by wastewater. Ryu et al. [45] showed that the matrix with oxidizing ability was effectively utilized by oxide metal ions under the action of H^+^ and microorganisms under reduced or flooded conditions in the SWIS. Therefore, it can be concluded that the system at the 95 cm matrix depth was mainly in an anaerobic environment, alternately transforming into anaerobic or anoxic environments occasionally.

### 3.3. Effects of Temperature on ORP

The experiment was performed in summer in Shenyang Province, and the temperature fluctuated only slightly in the laboratory, between 22 and 26 °C. As a result, the effects of temperature on ORP, Fe^2+^ and Mn^2+^ were neglected.

## 4. Conclusions

A novel SWIS was operated under three R_wd_s. The study demonstrated that ORP changes were non-linear with R_wd_s changing. ORP at 40 and 95 cm depths decreased during inflow periods and increased rapidly during dry periods. However, ORP showed inverse trends to those at the 65 cm depth. The optimal R_wd_ of the SWIS was 1:1. ORP at the 40 and 65 cm matrix depths from the inflow period to the dry period changed significantly, by 529 mV and 261 mV, at the R_wd_ of 1:1, which was considered to be beneficial for microbial activities. The content of Fe^2+^ and Mn^2+^ first increased and then decreased with the matrix depths increasing at three R_wd_s, which also further affected ORP. Nonlinear penetrations of ORP in the SWIS provided a satisfactory microenvironment for microorganisms. Moreover, the study suggested that the internal environment of the SWIS could transform from the aerobic state to the completely anaerobic state. Denitrification occurred when the wastewater was fed into the SWIS for 4 h. The SWIS with MDAS was capable of monitoring on-line ORP at different matrix depths simultaneously when intermittent operational conditions were changing, which was necessary in order to become more aquatinted with the internal environment of the SWIS in time for engineering applications. The SWIS had good ability to remove pollutants, and treated water met reuse requirements.

## Figures and Tables

**Table 1:** SWIS matrix properties.

Organic matter (%)	2.0
pH	6–9
Porosity (%)	55.4
Permeability (cm·s^−1^)	6.2 × 10^−4^
Hydraulic conductivity (cm·s^−1^)	(1.1 ± 0.5) × 10^−4^
>0.05 mm particle size distribution (%)	51.96
0.005–0.002 mm particle size distribution (%)	45.30
<0.002 mm particle size distribution (%)	2.24

**Table 2:** Inflow–outflow quality indicators for the SWIS.

	Inflow	Outflow
COD (mg·L^−1^)	82.5–153.2	11.3–36.4
BOD_5_ (mg·L^−1^)	51.7–96.6	4.9–11.2
NH_4_^+^-N (mg·L^−1^)	23.2–43.5	1.32–2.13
TN (mg·L^−1^)	30.1–50.4	13.6–19.7
TP (mg·L^−1^)	2.5–6.2	0.10–0.31
NO_3_^−^-N (mg·L^−1^)	3.27–15.70	6.93–17.45
NO_2_^−^-N (mg·L^−1^)	0.8–1.82	0.27–0.42
pH	7.17–7.60	7.12–7.46
Fe^2+^ (mg·L^−1^)	1.1–1.5	0.18–0.27
Mn^2+^ (mg·L^−1^)	0.31–0.53	0.07–0.1

COD: Chemical Oxygen Demand; TN: total nitrogen; TP: total phosphorous; BOD_5_: Biochemical oxygen demand

**Table 3:** Changes in Mn^2+^ content under wet-dry alternation operations at matrix depths at the R_wd_ of 1:1.

Matrix Depth (cm)	ORP (mV)	Mn^2+^ (mg·kg^−1^)	ORP (mV)	Mn^2+^ (mg·kg^−1^)
145	−409	6.53	−409	3.64
95	−124	7.04	−353	5.62
65	232	3.91	389	10.06
40	514	2.52	479	1.24
25	502	0.64	505	0.89
